# Case report and brief literature review: possible association of secukinumab with Guillain–Barré syndrome in psoriasis

**DOI:** 10.3389/fimmu.2024.1412470

**Published:** 2024-06-28

**Authors:** Gang Liang, Yongmei Han, Haiyan He, Ci Lu, Chen Zhu

**Affiliations:** ^1^ Department of Pharmacy, Sir Run Run Shaw Hospital, School of Medicine, Zhejiang University, Hangzhou, China; ^2^ Department of Rheumatology, Sir Run Run Shaw Hospital, School of Medicine, Zhejiang University, Hangzhou, China

**Keywords:** adverse drug reaction (ADR), secukinumab, Guillain–Barré syndrome (GBS), case report, IL-17

## Abstract

The etiology of Guillain–Barré syndrome (GBS) may be autoimmune. About two-thirds of patients typically experience their first symptoms within 5 days to 3 weeks after common infectious diseases, surgery, or vaccination. Infection is a triggering factor for over 50% of patients. In recent years, a growing number of studies have indicated that some immune checkpoint inhibitors and COVID-19 may also contribute to the occurrence of GBS. However, drugs are considered a rare cause of GBS. The patient in our case was a 70-year-old man who developed GBS after initiating secukinumab for psoriasis. Upon diagnosis suggesting a potential association between secukinumab and the development of GBS, as per the Naranjo adverse drug reaction (ADR) probability scale, we decided to discontinue the drug. Following this intervention, along with the administration of immunoglobulin, the patient exhibited a significant improvement in extremity weakness. The association of GBS with secukinumab treatment, as observed in this case, appears to be uncommon. The underlying mechanisms that may link secukinumab to the development of GBS are not yet fully understood and warrant further scientific inquiry and rigorous investigation. However, we hope that this report can raise greater awareness and vigilance among medical professionals to enhance the safety of patients’ medication.

## Introduction

Secukinumab is a fully human anti–Interleukin-17A (IL-17A) G1k monoclonal antibody that selectively targets and neutralizes IL-17A (1). Based on the pivotal phase III randomized controlled trial in China (2), secukinumab was approved for the treatment of adult patients with moderate-to-severe plaque psoriasis by the China National Medical Products Administration in 2019. Later, Avallone et al. underscored the remarkable efficacy and safety profile of IL-17 antagonists in managing difficult-to-treat forms of psoriasis, notably pustular psoriasis (3). The most common adverse events (AEs) of secukinumab were nasopharyngitis, superficial skin bacterial infection, urticaria, and upper respiratory infection (4). We report a case of Guillain–Barré syndrome (GBS) following the initiation of secukinumab for psoriasis. According to reports, several drugs can induce GBS, including tumor necrosis factor–alpha (TNF-α) antagonists (5), tacrolimus (6), isotretinoin (7), and immune checkpoint inhibitors (ICIs) (8–11). To the best of our knowledge, there have been no report documenting a direct causal relationship between secukinumab and the development of GBS.

## Case presentation

**Figure 1:**
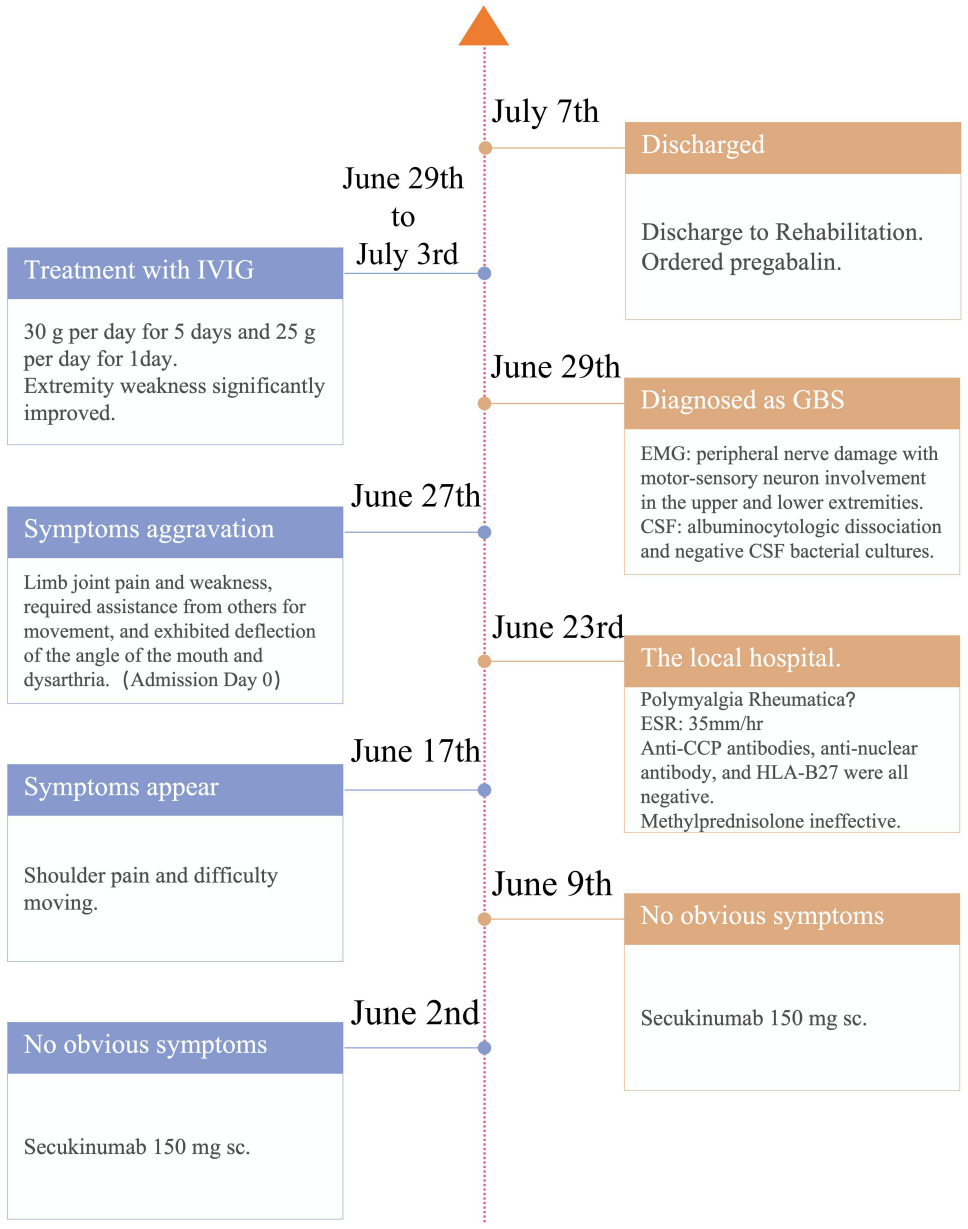
Timeline of the case report. sc, subcutaneous injection; ESR, erythrocyte sedimentation rate; HLA-B27, human leukocyte antigen B27; GBS, Guillain–Barré syndrome; EMG, electromyography; CSF, cerebrospinal fluid; IVIG, intravenous immunoglobulin.

A 70-year-old male patient has been experiencing a dotted or patchy rash, partially covered with white scales, for 40 years. He has been treated with a “hormone-containing ointment” (unclear). He received a subcutaneous injection of secukinumab at 150 mg twice (150 mg per week) without using topical ointments. In the meantime, psoriasis remarkably improved. After 8 days of the second administration, the patient experienced shoulder pain and difficulty moving. Subsequently, they gradually developed pain below the knees in both lower extremities, as well as pain in the proximal and distal interphalangeal joints of both hands, along with bilateral lower extremity weakness. At the beginning, the patient was still able to walk slowly but, later, required assistance from others for movement. Additionally, the patient exhibited deflection of the angle of the mouth and dysarthria. The physical examination upon admission showed normal body temperature, blood pressure, pulse, respiratory rate, and oxygen saturation, with a pain score of 4. Muscle weakness affected all his extremities, with a severe decrease in strength graded at 1–2. Rheumatoid factor, anti-Cyclic Citrullinated Peptide (CCP) antibodies, anti-nuclear antibody, and human leukocyte antigen B27 were all negative at another hospital. After admission, the routine examinations showed that the serum potassium, creatine kinase, and C-reactive protein levels were within the normal range. Computed tomography of the head was unremarkable. Electromyography, however, demonstrated peripheral nerve damage with motor-sensory neuron involvement in the upper and lower extremities. The cerebrospinal fluid (CSF) analysis demonstrated a significant albuminocytologic dissociation, characterized by a markedly elevated protein level of 1563 mg/L, exceeding the normal range of 150 mg/L to 450 mg/L, with a positive Pandy’s test, alongside a normal cell count. Additionally, CSF bacterial cultures were negative. Further testing, encompassing acid-fast staining, India ink staining, and Gram staining all returned negative results. The antibody test for herpes virus also showed a negative outcome. Based on the results above, the patient was diagnosed with GBS. Extremity weakness significantly improved after the administration of immunoglobulin (30 g/day for 5 days and 25 g/day for 1 day). The timeline of the case was illustrated in **Figure 1**. The patient was compliant and expressed understanding with all treatment received. According to the Naranjo adverse drug reaction (ADR) probability scale, the probability of secukinumab-induced GBS in this case is classified as probable (**Table 1**).

**Table 1:** Naranjo adverse drug reaction probability scale.

Question	Yes	No	Do Not Know	Score
1. Are there previous conclusive reports on this reaction?	+1	0	0	0
2. Did the adverse event appear after the suspected drug was administered?	+2	−1	0	+2
3. Did the adverse event improve when the drug was discontinued or a specific antagonist was administered?	+1	0	0	+1
4. Did the adverse event reappear when the drug was readministered?	+2	−1	0	0
5. Are there alternative causes that could on their own have caused the reaction?	−1	+2	0	+2
6. Did the reaction reappear when a placebo was given?	−1	+1	0	0
7. Was the drug detected in blood or other fluids in concentrations known to be toxic?	+1	0	0	0
8. Was the reaction more severe when the dose was increased or less severe when the dose was decreased?	+1	0	0	0
9. Did the patient have a similar reaction to the same or similar drugs in any previous exposure?	+1	0	0	0
10. Was the adverse event confirmed by any objective evidence?	+1	0	0	0
	Total score: 5			

## Discussion

The etiology of GBS may be autoimmune. About two-thirds of patients typically experience their first symptoms within 5 days to 3 weeks after contracting ordinary infectious diseases, undergoing surgery, or receiving a vaccination. Infection is a triggering factor for over 50% of patients and the most common pathogens include *C. jejuni*, *enteroviruses*, *herpesvirus*, and *mycoplasma*. In recent years, a growing number of studies have indicated that some ICIs (12, 13) and *COVID-19* (14, 15) may also contribute to the development of GBS. However, all the above reasons were ruled out in this patient, and only a small amount of evidence suggests that psoriasis is associated with GBS. Based on the above considerations and the Naranjo scale, secukinumab probably induced GBS in the present patient. Although there have been individual cases of peripheral nervous system (PNS) disorders, including GBS, caused by drugs related to the IL-17 pathway (such as ixekizumab (16, 17) and ustekinumab (18)), this paper reports a potential association between secukinumab treatment and the development of GBS, which, to our knowledge, has not been previously documented.

In 2018, Osman et al. (18) first reported a case of ustekinumab-related chronic inflammatory demyelinating polyneuropathy. Scholars in Ireland (16) and China (17) have, in their studies from 2021 and 2023, respectively, identified a link between ixekizumab and the onset of multifocal motor neuropathy and GBS. However, the mechanism by which IL-17 pathway inhibitors induce PNS disorders remains unknown. Three reported cases mentioned above (16–18) suggested the complexity of cytokine networks in the pathogenesis of PNS disorders. The use of IL-17 pathway inhibitors may disrupt the balance in the cytokine network, potentially leading to the development of PNS disorders, such as GBS. Cytokine networks in the pathogenesis of GBS could be classified into three immune axes (19): the Th0–Th1 immune axis, the Th0–Th17 immune axis, and the Th0–Th2 immune axis. These axes include pro-inflammatory cytokines (IL-1β, IL-6, IL-12, IL-17, IL-18, IL-23, TNF-α, interferon-γ (IFN-γ), and chemokines) as well as anti-inflammatory cytokines (IL-4, IL-10, and transforming growth factor-β (TGF-β)) (20). When the balance is disrupted, demyelinating neuropathies may occur. Gelato and Mastorino, in their analysis of a patient who developed multiple sclerosis (MS) after starting dupilumab treatment for atopic dermatitis, hypothesized that dupilumab could induce a shift toward Th1/Th17 polarization by blocking the shared receptor subunit for IL-4 and IL-13. This alteration in immune response could, in turn, trigger or worsen Th1/Th17-associated conditions such as psoriasis and MS in genetically predisposed individuals (21). If this is the case, then secukinumab might have a role in the therapy of GBS, and the same point of view was also raised in some other studies (19, 22). Unexpectedly, sometimes, the loss of IL-17A did not diminish the pathogenicity of Th17 cells in neuroinflammatory disease (23). Drawing from the existing body of research, we have distilled two potential scenarios. One possibility is the activation of IL-17R signaling that occurs independently of IL-17. This involves a molecular complex that includes the adaptor molecule Act1 and the tyrosine phosphatase Src homology-2 domain-containing protein tyrosine phosphatase-2 (SHP2), which mediates autonomous IL-17R signaling, thereby accelerating and sustaining inflammation (24). Another possibility is highlighted by Chong et al. (23), who discovered that IL-17A plays a dual role in Th17 cells, functioning both as a pathogenic and a regulatory factor. The cytokine IL-17A limits Th17 pathogenicity by primarily repressing IL-17F and Granulocyte Macrophage-Colony Stimulating Factor (GM-CSF) through a negative feedback loop driven by autocrine induction of IL-24. Silencing of IL-24 in Th17 cells, caused by IL-17A antagonists, enhances disease progression. However, in our case study, we were unable to measure the cytokine levels in the CSF both prior to and at the time of symptom onset, which precludes the verification of the pathogenic mechanism.

In the present case, the patient has not used biological agents since the onset of treatment and is currently receiving only medications for symptom relief, specifically pregabalin and mecobalamin, while also maintaining ongoing consultations with the rehabilitation department. From the published cases of GBS caused by IL-17 antagonists or TNF-α antagonists, there was a possibility that two treatment regimens were switched with no GBS recurrence (17, 22). However, we considered more data, and long-term monitoring of these patients will be needed to confirm the safety of these regimens.

## Conclusion

In summary, GBS occurring in the context of IL-17 pathway inhibition represents an infrequent ADR, and the possible mechanisms need to be further explored. We hope that this report can raise greater awareness and vigilance among the majority of medical personnel to enhance the safety of patients’ medication.

## Data availability statement

The original contributions presented in the study are included in the article/supplementary material. Further inquiries can be directed to the corresponding author.

## Ethics statement

The studies involving humans were approved by Ethics Committee of Sir Run Run Shaw Hospital. The studies were conducted in accordance with the local legislation and institutional requirements. Written informed consent for participation was not required from the participants or the participants’ legal guardians/next of kin in accordance with the national legislation and institutional requirements. Written informed consent was obtained from the participant/patient(s) for the publication of this case report.

## Author contributions

GL: Writing – review & editing, Writing – original draft, Formal analysis. YH: Writing – original draft, Formal analysis. HH: Writing – review & editing, Methodology. CL: Writing – review & editing, Formal analysis, Data curation. CZ: Writing – original draft, Writing – review & editing, Methodology, Conceptualization.
